# Frequent activating STAT3 mutations and novel recurrent genomic abnormalities detected in breast implant-associated anaplastic large cell lymphoma

**DOI:** 10.18632/oncotarget.26308

**Published:** 2018-11-16

**Authors:** Piers Blombery, Ella Thompson, Georgina L. Ryland, Rachel Joyce, David J. Byrne, Christine Khoo, Stephen Lade, Mark Hertzberg, Greg Hapgood, Paula Marlton, Anand Deva, Geoffrey Lindeman, Stephen Fox, David Westerman, Miles Prince

**Affiliations:** ^1^ Department of Pathology, Peter MacCallum Cancer Centre, Melbourne, VIC, Australia; ^2^ Sir Peter MacCallum Department of Oncology, The University of Melbourne, Parkville, VIC, Australia; ^3^ Stem Cells and Cancer Division, The Walter and Eliza Hall Institute of Medical Research, Parkville, VIC, Australia; ^4^ Department of Medical Biology, The University of Melbourne, Parkville, VIC, Australia; ^5^ Department of Haematology, Prince of Wales Hospital, University of New South Wales, Randwick, NSW, Australia; ^6^ Department of Haematology, Princess Alexandra Hospital, Brisbane, QLD, Australia; ^7^ Surgical Infection Research Group, Faculty of Medicine and Health Sciences, Macquarie University, Sydney, NSW, Australia; ^8^ Department of Medicine, The University of Melbourne, Parkville, VIC, Australia

**Keywords:** lymphoma, genomics, NGS

## Abstract

Breast implant-associated anaplastic large cell lymphoma (BIA-ALCL) is a rare form of T-cell lymphoma that occurs after implantation of breast prostheses. We performed comprehensive next generation sequencing based genomic characterization of 11 cases of BIA-ALCL including sequence variant detection on 180 genes frequently mutated in haematological malignancy, genome-wide copy number assessment, structural variant detection involving the T-cell receptor loci and TRB deep-sequencing. We observed sequence variants leading to JAK/STAT activation in 10 out of 11 patients. We also observed germline *TP53* mutations in two cases. In addition we detected a recurrent copy number loss involving RPL5 as well as copy number amplifications involving *TNFRSF11A* [RANK] (in 2 cases), *MYC*, *P2RX7*, *TMEM119* and *PDGFRA*. In summary, our comprehensive genomic characterisation of 11 cases of BIA-ALCL has provided insight into potential pathobiological mechanisms (JAK/STAT, MYC and TP53) as well as identifying targets for future therapeutic intervention (TNFRSF11A, PDGFRA) in this rare entity.

## INTRODUCTION

Breast implant-associated anaplastic large cell lymphoma (BIA-ALCL) is a rare form of T-cell lymphoma that occurs after implantation of breast prostheses typically after a relatively long latency [1, 2]. BIA-ALCL is notable for its aggressive histological appearance but a paradoxical predominance of clinical presentation with early stage disease and a relatively favourable prognosis when compared with its systemic counterpart [3, 4]. Whilst the underlying cause of BIA-ALCL is unknown, a possible contribution from chronic antigen stimulation by a unique implant-biofilm associated microbiome has been hypothesized [5].

Genomic and functional characterization of systemic ALK-negative anaplastic large cell lymphoma (sALCL) has revealed the importance of STAT3 activation, MYC expression, *PRDM1*/*TP53* abnormalities and recurrent structural variants involving the *DUSP22* and *TP63* loci [6–9]. By contrast, the genomic landscape of BIA-ALCL and its relevant pathogenic drivers are significantly less well characterized. Whole exome sequencing of two cases and targeted sequence variant detection of case series to date have demonstrated activating mutations in the JAK/STAT pathway in a proportion of cases [10–12]. We aimed to extend the understanding of this rare lymphoma by performing comprehensive genomic characterization by targeted sequence variant detection, whole genome copy number assessment, T-cell receptor locus structural variant detection and T-cell receptor repertoire sequencing on a cohort of cases of BIA-ALCL.

## RESULTS

### Patient cohort

Thirteen patients were referred for diagnostic NGS in the study period as part of investigation of newly diagnosed BIA-ALCL. Eleven out of thirteen patients had sufficient quality and quantity of DNA for NGS analysis. All eleven patients were female and the median age of the cohort was 42 years (range 29–59). Eight patients had stage IA (T1N0M0), two patients had stage IB (T2N0M0) and one patient had stage IIA (T4N0M0) disease by MD Anderson TNM staging [13].

### Sequence variant detection

Pathogenic sequence variants detected in the eleven cases are listed in Table 1. Pathogenic activating *STAT3* mutations were detected in 7 out of 11 cases (64%). In one of the four *STAT3* wildtype patients, we detected a truncating mutation of *SOCS1* which is predicted to result in activation of the JAK/STAT pathway through loss of negative regulation [14]. Another *STAT3* wildtype patient had an activating *JAK1* mutation detected as previously described [10]. Finally, one *STAT3* wildtype patient (BALCL11) had a novel sequence variant detected in *PTPN1* (Thr263Ile). *PTPN1* encodes PTP1B which dephosphorylates tyrosine residues and through this mechanism is involved in regulation of intracellular signalling pathways including the JAK/STAT pathway [15]. This variant causes a change in an amino acid which is critical for protein function and is predicted to be deleterious by multiple *in silico* predictors [16]. Loss of PTPN1 function through mutation has been shown to result in JAK/STAT activation in primary mediastinal B-cell lymphoma and Hodgkin lymphoma [17]. Therefore, in ten out of eleven cases (91%) we observed direct evidence of JAK/STAT activation attributable to sequence variants alone.

**Table 1:** Sequence variant, copy number changes and TRB characteristics from eleven cases of breast implant associated anaplastic large cell lymphoma (BIA-ALCL)

Sample ID	Stage of Disease	Gene	HGVSc	HGVSp	VAF	Focal copy number loss	Focal copy number gain	TCR VDJ	CDR3 AA
**BALCL1**	T2N0M0	STAT3	NM_139276.2:c.1981G>T	p.(Asp661Tyr)	23.5%	PRDM1, PTPN1, RPL5	Nil	TRBV5-1/TRBD1/TRBJ1-2	CASSLGHQLNYGYTF
		BCOR	NM_017745.5:c.4424G>A	p.(Trp1475^*^)	20.7%				
**BALCL2**	T1N0M0	STAT3	NM_139276.2:c.1919A>T	p.(Tyr640Phe)	56.1%	Nil	TNFRSF11A [RANK] (~ 21 copies)	TRBV14/TRBD1/TRBJ1-6	CASATSTLYNSPLHF
**BALCL3**	T4N0M0	TP53^*^	NM_000546.5:c.673-1G>A	p.?	50.0%	Nil	Nil	TRBV11-2/TRBJ2-2	CASSPRAPNTGELFF
**BALCL4**	T1N0M0	SOCS1	NM_003745.1:c.518dup	p.(Leu174Alafs^*^79)	37.8%	SETD2, RPL5	Nil	TRBV13/TRBD2/TRBJ1-1	CASSLGWGGGSEAFF
**BALCL5**	T1N0M0	STAT3	NM_139276.2:c.1981G>T	p.(Asp661Tyr)	42.6%	PRDM1, RPL5	KIT/PDGFRA (~6 copies), TNKS(~8 copies),	TRBV30/TRBJ2-4	CAWANWGNIQYF
**BALCL6**	T2N0M0	TP53	NM_000546.5:c.524G>A	p.(Arg175His)	23.8%	PRDM1, PML (hom), BCORL1 (hom)	MYC (~10 copies)	TRBV12-4/TRBJ1-1	CASSFRQTEAFF
		STAT3	NM_139276.2:c.1229A>G	p.(His410Arg)	24.8%				
		TP53^*^	NM_000546.5:c.746G>A	p.(Arg249Lys)	23.6%				
		SETD2	NM_014159.6:c.2893G>T	p.(Glu965^*^)	44.3%				
**BALCL7**	T1N0M0	STAT3	NM_139276.2:c.1840A>C	p.(Ser614Arg)	59.5%	RPL5	Nil	TRBV30/TRBD2/TRBJ1-1	CAWGIGGGEAFF
**BALCL8**	T1N0M0	JAK1	NM_002227.2:c.3290_3291delinsTT	p.(Gly1097Val)	20.0%	Nil	Nil	TRBV11-1/TRBD1/TRBJ2-1	CASSGSGNHEQFF
		JAK3^*^	NM_000215.3:c.2164G>A	p.(Val722Ile)	46.3%				
**BALCL9**	T1N0M0	STAT3	NM_139276.2:c.1981G>T	p.(Asp661Tyr)	29.0%	Nil	Nil	TRBV18/TRBJ2-3	CASSPLGGEDTQYF
**BALCL10**	T1N0M0	STAT3	NM_139276.2:c.1842C>A	p.(Ser614Arg)	8.2%	Nil	TNFRSF11A [RANK] (~6 copies)	TRBV5-4/TRBD1/TRBJ2-6	CASSLGGSAGANVLTF
**BALCL11**	T1N0M0	PTPN1	NM_002827.3:c.788C>T	p.(Thr263Ile)	72.5%	RPL5	P2RX7 (~7 copies), TMEM119 (~49 copies)	TRBV5-1/TRBJ1-1	CASSLGATGTEAFF
		PRKCB	NM_212535.2:c.1265A>G	p.(Tyr422Cys)	40.3%				

^*^confirmed germline, Abbreviations: hom – homozygous, VAF (variant allele frequency).

Patient BALCL11 also had an acquired sequence variant detected in *PRKCB* (Tyr422Cys). This is a previously undescribed variant that results in an amino acid change in a residue which stabilizes the interaction between PRKCB and diacyglycerol (DAG). Mutations in similarly critical residues have been shown to result in protein kinase β (PKCβ) pathway activation in adult T-cell leukemia/lymphoma (ATLL) [18].

Three pathogenic *TP53* variants were detected in two patients. Both patients with *TP53* mutations had breast implants inserted after undergoing mastectomy for breast carcinoma. Moreover, both patients had a positive family history of breast cancer. Subsequent testing of germline samples from these patients confirmed the germline origin of the two *TP53* variants (c.673-1G>A and Arg249Lys) identified in both cases, BALCL3 and BALCL6 respectively. The *TP53* c.673-1G>A mutation affects the canonical splice on the intron 6/exon 7 boundary with functional validation of a splicing defect and has been observed in multiple Li-Fraumeni kindreds [19–21]. The *TP53* Arg249Lys mutation has not been described in Li-Fraumeni kindreds, however it has been observed multiple times as an acquired variant in diverse malignancies as well as being categorised as non-functional in the *TP53* IARC database [22].

### Copy number assessment

We focussed our genome wide copy number analysis on recurrent abnormalities (i.e. those occurring in more than one patient) as well as focal (generally <5 Mb or involving single coding genes) deletions and amplifications as these are most likely to provide insights into lymphoma biology. Recurrent and focal copy number changes are shown in Table 1.

**Figure 1:**
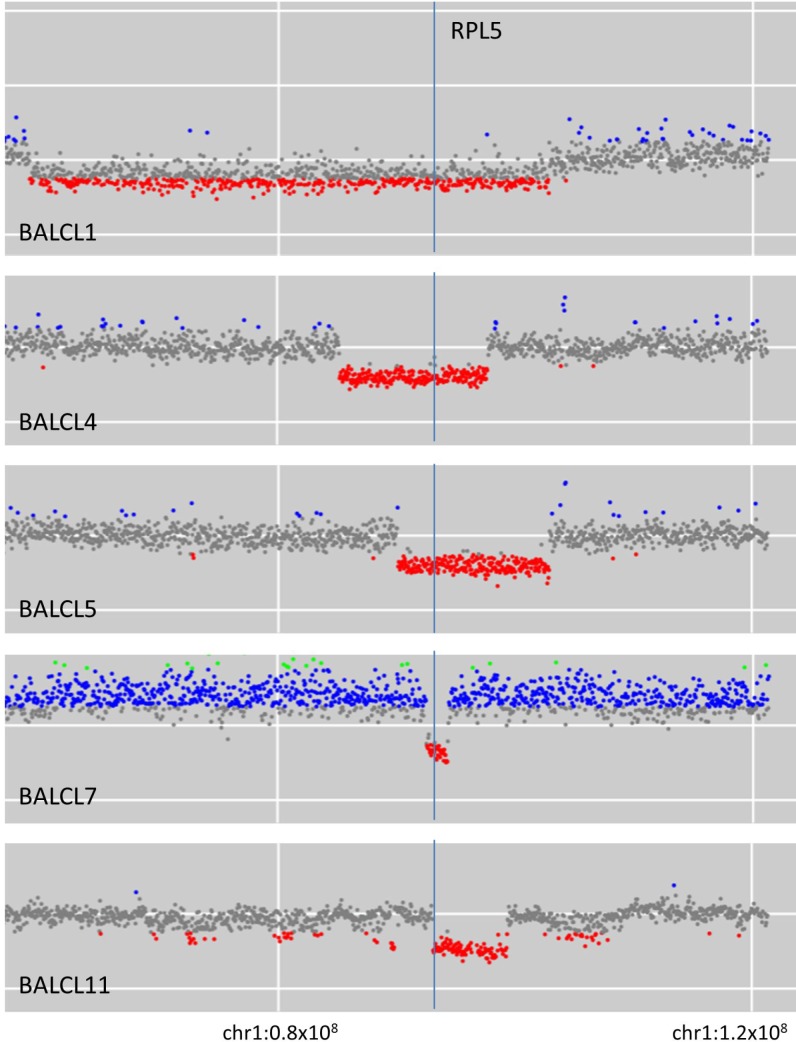
Five cases of breast implant-associated anaplastic large cell lymphoma with deletion on chromosome 1p involving *RPL5*

A recurrent focally deleted region on chromosome 1p was observed in five cases. Deletions in these five cases involved 1p21-22 with a minimal deleted region (MDR) between nucleotides 92340000 and 94450000 on chromosome 1 (human reference sequence GRCh37 [hg19]; Figure 1). This MDR contains the recently characterised haploinsufficient tumor suppressor gene *RPL5* [23].

*TP53* and *PRDM1* (encoding BLIMP-1) loci were specifically assessed due to the presence of recurrent losses in sALCL [24]. *PRDM1* copy number loss was observed in three out of eleven cases (27%) but no *TP53* copy number losses were detected.

**Figure 2:**
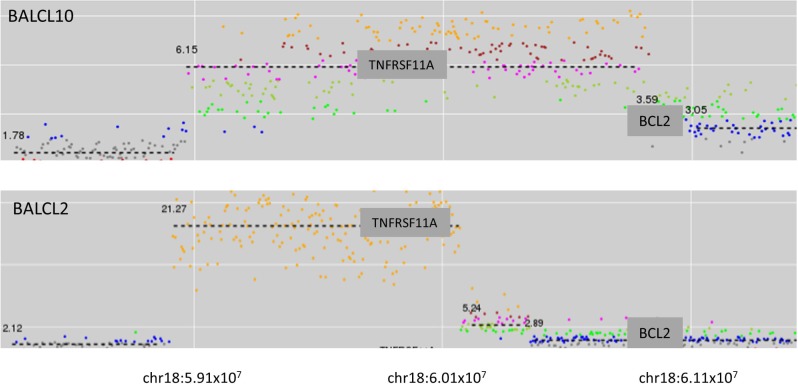
Two cases of breast implant-associated anaplastic large cell lymphoma with high level amplification of *TNFRSF11A* (RANK)

**Figure 3:**
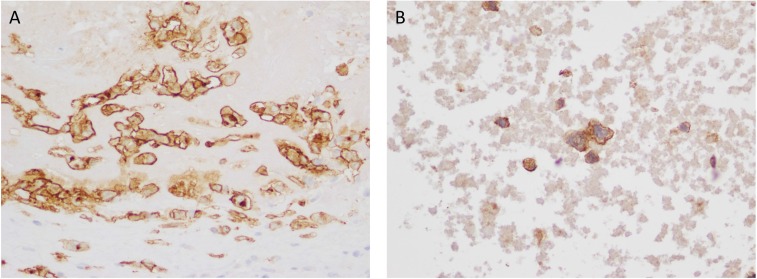
(**A**) RANK immunohistochemistry in a case of breast implant-associated anaplastic large cell lymphoma (BIA-ALCL) with TNFRSF11A (RANK) amplification (×400) (**B**) PDGFRA immunohistochemistry in a case of BIA-ALCL with PDGFRA amplification.

Focal high-level amplifications were detected in five cases. In two of these cases the focal amplification involved 18q21 (Figure 2). In both cases the amplified genomic segment involved *TNFRSF11A* (which encodes Receptor Activator of Nuclear Factor κB [RANK]). Immunohistochemistry (IHC) staining for RANK was performed on one of the cases where formalin-fixed, paraffin-embedded (FFPE) tumor material was available (BALCL10). Strong membrane staining of tumor cells was observed (Figure 3A).

In one case a high level focal amplification on 4q12 involving both *PDGFRA* and *KIT* was detected. IHC for PDGFRA showed strong membrane staining of tumor cells (Figure 3B). This patient also had a focal amplification of 8p23.1 involving *TNKS,* the gene encoding Tankyrase – a poly ADP-ribose polymerase (PARP) enzyme involved in Wnt/β-catenin pathway.

In BALCL11 a copy number amplification (approximately 50 copies) was detected involving *TMEM119*, a recently characterised oncogene implicated in transforming growth factor beta (TGF-β) signalling in osteosarcoma [25]. This patient also had a copy number amplification (approximately 7 copies) involving *P2RX7* which encodes the P2×7 purine-receptor and which is associated with complex immune effects including involvement in NLRP3 inflammasome assembly, T-cell survival and differentiation [26].

**Figure 4:**
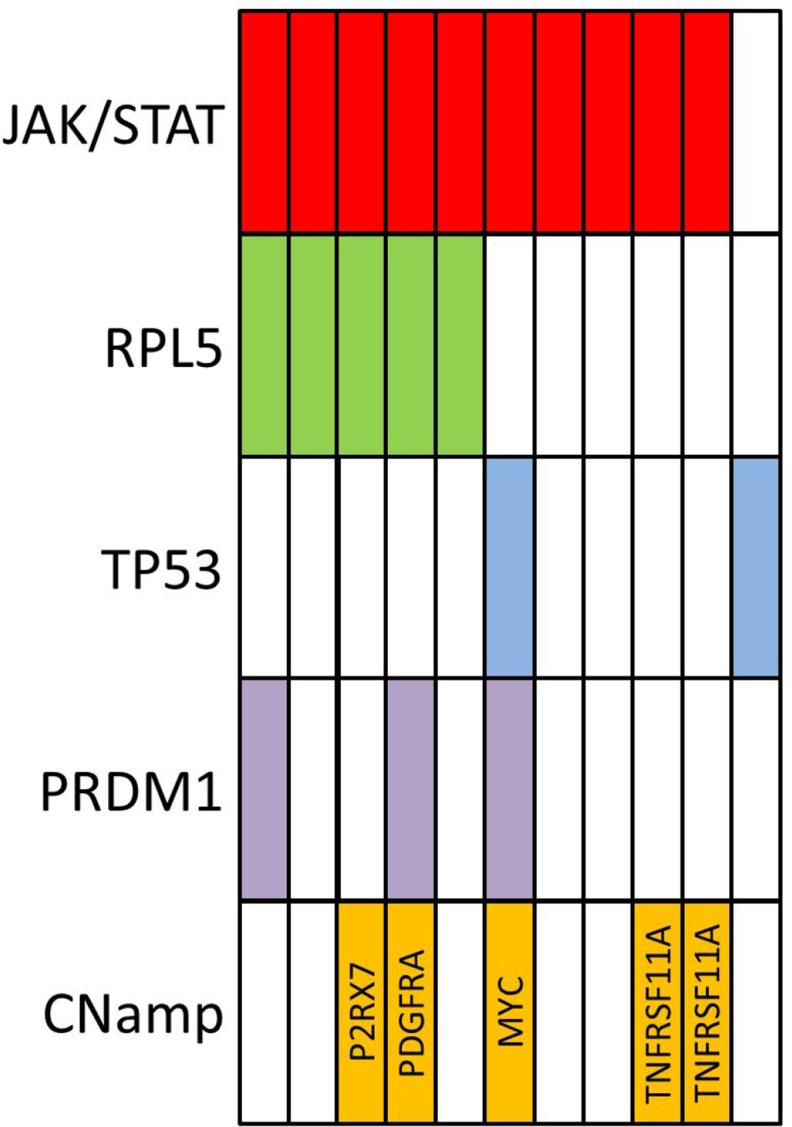
Summary of recurrent genomic findings from 11 cases of breast implant-associated large lymphoma (CNamp–copy number amplification)

A focal amplification of MYC (approximately 10 copies) was observed in BALCL6. No structural variants involving the *TRA*, *TRB*, *TRG* or *TRD* loci were detected. A summary of recurrent genomic abnormalities are shown in Figure 4.

### T-cell repertoire sequencing

*TRB* amplicon deep sequencing was performed on all patients. A dominant clonal rearrangement was detected in *TRB* in all cases. The VDJ family usage and CDR3 amino acid sequences for *TRB* sequencing are shown in Table 1. The CDR3 sequences of the dominant clones were queried against a curated database of TCR sequences associated with known antigen specificity using VDJdb [27]. The dominant clonal *TRB* sequences were not predicted to have affinity with known antigens in the VDJdb database. Seroma T-cell repertoire was analysed after removal of the dominant clonal sequence (i.e. leaving residual sequences representing the non-malignant T-cell infiltrate [confirmed through different V/D/J-family usage]). The non-malignant seroma T-cell repertoire was found to have decreased diversity when compared to normal polyclonal T-cells obtained from peripheral blood of healthy volunteers (normalized Shannon Index 0.843 vs 0.959, *p* = 0.0044).

## DISCUSSION

We have described the genomic findings of a cohort of BIA-ALCL which has provided insights into the pathobiology of this rare entity. Aberrant STAT3 signalling is a central pathogenic abnormality in sALCL and has also been observed to be strongly activated in BIA-ALCL cell lines [6, 28, 29]. There are multiple genomic routes through which STAT3 activation can be achieved in sALCL including activating sequence variants in *JAK1*/*STAT3* (in approximately 20–30% of cases) and translocations involving *ROS1* and *TYK2* [6]. In contrast, we observed direct evidence of JAK/STAT activation attributable to sequence variants alone in ten out of eleven cases in our BIA-ALCL cohort - predominantly through activating *STAT3* mutations (in seven patients). Therefore whilst aberrant STAT3 activation appears to be ubiquitous in both in BIA-ALCL and sALCL, the genomic mechanism by which this signalling aberration is achieved appears to be different. Moreover, the spectrum of *STAT3* mutations observed in our cohort also differed from those previously described in sALCL [6] with three of the seven *STAT3* mutations occurring outside the typical Tyr640 (and surrounding codons). We observed two *STAT3* Ser614Arg mutations as well as one mutation in the DNA-binding domain (*STAT3* His410Arg). The explanation for the predilection of JAK/STAT3 activation in BIA-ALCL through STAT3 activating mutations is not clear. Whilst JAK1/STAT3 sequence variants have previously been described in BIA-ALCL, the frequency in our cohort is significantly higher than previously observed [11, 30].

BIA-ALCL has been described in the context of pathogenic germline *TP53* abnormalities in two separate case reports to date [31, 32]. In our cohort, we observed two further cases with confirmed pathogenic germline *TP53* mutations. Both these patients had breast implant insertion after surgery for breast cancer and had a family history of breast cancer. In one of these cases a second *TP53* mutation (Arg175His) was acquired in the patient's tumor consistent with Knudson's two-hit paradigm of tumorigenesis. Despite the description of four cases in total to date with germline *TP53* abnormalities, the overall number of patients with germline *TP53* mutations undergoing breast implant insertion is unclear and therefore the precise risk in this population cannot be determined. However given the observed association to date and the increase in risk of these patients to a variety of malignancies it is a phenomenon that warrants further monitoring and study.

We have described a novel recurrent copy number loss on chromosome 1p in our cohort of BIA-ALCL. The minimally deleted region occurring at 1p22 was approximately 2 Mb in size and is the same area that has been recently observed to be recurrently deleted in multiple myeloma [33]. Experimental evidence and investigation of deletions in this area have indicated that the most likely coding gene target of the deletion is *RPL5*, a gene encoding a ribosomal protein which forms part of the 60S ribosomal subunit and which shows decreased expression in deleted cases [23, 33]. Evidence to date suggests that *RPL5* (along with numerous other ribosomal protein genes [RPGs]) is a haploinsufficient tumor suppressor gene which is frequently deleted in a range of cancers including glioblastoma multiforme, melanoma and breast cancer [23]. In a large cancer dataset analysis, RPG deletions were associated with *TP53* mutation and/or *TP53* copy number loss [34]. It has been hypothesized that deletion of RPGs (such as *RPL5*) results in ribosomal biogenesis stress which triggers TP53 activation and cell death, therefore a selection against these deletions in the face of an intact TP53 pathway has been proposed [34]. Interestingly in our cohort, we detected *RPL5* deletions exclusively in patients that were *TP53* wildtype by mutational analysis and copy number (Figure 4). Although this may seem contradictory to the negative selection hypothesis, in the majority of cases of sALCL it has been observed that the TP53 pathway is abnormal as assessed by positive TP53 immunohistochemical staining, however, this is typically not due to recurrent mutation or copy number loss [35]. Therefore, ALCL may represent a permissive environment for *RPL5* deletion due to the high prevalence of multifactorial TP53 dysfunction. Of note, abnormal TP53 activation in response to DNA damage by radiation and cytotoxic agents has been observed in BIA-ALCL cell lines [36]. Whilst the rate of TP53 pathway abnormalities in BIA-ALCL is not known, the observation of two patients with germline *TP53* mutations and recurrent deletion in *RPL5* is supportive evidence of the biological importance of an aberrant TP53 pathway in BIA-ALCL.

In addition, RPL5 has been shown to be involved in suppression of MYC expression [37] and RPL5 knockdown is associated with increased MYC expression in breast cancer cell lines [23]. MYC dysregulation (driven by IRF4 signalling) has been recognised as a central pathogenic abnormality in sALCL [7]. The observation of recurrent *RPL5* deletions in our cohort as well as the observation of a high-level *MYC* copy number gain observed in BALCL6 provides evidence of the potential importance of *MYC* aberration in this disease.

We also detected novel focal high level amplifications involving multiple oncogenes of potential relevance to lymphoma pathogenesis including *TNFRSF11A*, *PDGFRA*, *TMEM119* and *P2RX7*. *TNFRSF11A* encodes Receptor Activator of Nuclear Factor κ B (RANK) and ligation of this receptor by RANKL results in NF-κB activation. RANKL plays a role in normal breast physiology, where it is produced by mammary cells in response to progesterone and has been shown to be a key paracrine factor that activates mammary stem/progenitor cells [38, 39]. The RANKL/RANK signaling pathway has also been implicated in progesterone-mediated breast tumorigenesis and *BRCA1*-mutated breast cancer [40, 41]. In metastatic breast cancer, tumor infiltrating regulatory T-cells (Treg) [42] may also contribute to local RANKL production. Thus the breast is a recognized tissue responsible for local RANKL secretion, where RANKL/RANK signalling (which activates the NF-κB pathway) induces pro-survival and growth signals. Invariant NKT cells also contribute to RANKL production in the microenviroment in multiple myeloma [43]. The finding that RANK can be over-expressed in BIA-ALCL has potential therapeutic implications, given the availability of monoclonal antibody RANKL inhibitors that are currently in clinical use for the treatment of osteoporosis and prevention of skeletal related events in metastatic breast cancer [44, 45].

The observation of *PDGFRA* amplification and confirmed overexpression is of interest given that, in ALK+ sALCL, ALK has been shown to induce the expression of PDGFR via JUNB and JUN which subsequently contributes to the malignant behaviour of ALK+ ALCL [46]. Moreover, inhibition of PDGFRA and PDGFRB by imatinib has been used in a patient with refractory ALK+ ALCL with reported efficacy [46]. The detection of *PDGFRA* amplification and overexpression in our patient supports PDGFRA as another potentially targetable lesion.

It is well-established that the majority of cases of ALK-negative sALCL do not express a T-cell receptor despite having rearranged *TRG* and *TRB* loci [47]. We sequenced *TRB* in our cohort and show that, similar to ALK-negative sALCL, all cases of BIA-ALCL harboured a rearranged *TRB* locus. One hypothesis of BIA-ALCL development is chronic antigen stimulation by microorganisms contained in a biofilm over the implant. Whilst our observation of restricted diversity in the non-malignant T-cell compartment is supportive of this hypothesis, this could be further investigated more directly with stimulation of BIA-ALCL cells with candidate antigens.

In summary, we have comprehensively genomically characterized eleven cases of BIA-ALCL. Our genomic data demonstrate that the aberrant signalling pathways in BIA-ALCL are analogous to those in sALCL including abnormalities of the TP53, MYC and JAK/STAT3 pathways. However, the observed set of genomic abnormalities that achieve dysregulation of these pathways appears to be unique in BIA-ALCL with highly recurrent activating *STAT3* mutations and recurrent deletions of 1p22 involving *RPL5* which have not been identified in sALCL to date. In addition, our genomic data also implicates abnormalities of TGF-β, PKC, Wnt/β-catenin pathway and inflammosome signalling as areas of future study. Finally, we have made the important observation of high-level amplification of *TNFRSF11A* and *PDGFRA* in a proportion of cases which are potentially targetable by available therapeutics. These findings provide a basis for future functional studies to clarify the roles of the pathways implicated by these genomic aberrations in the pathogenesis of BIA-ALCL.

## MATERIALS AND METHODS

### Patient cohort

Consecutive cases of BIA-ALCL were identified from July 2015 to July 2018 that had been referred for diagnostic molecular testing at the Peter MacCallum Cancer Centre Molecular Haematology Laboratory (Melbourne, Australia). All patients consented for genomic testing as part of their tumor diagnostic workup as per institutional guidelines, as well as with approval by the ethics committee of the Peter MacCallum Cancer Centre (03/09) and research was conducted in accordance with the Helsinki Declaration of 1975, as revised in 2008. The diagnosis of BIA-ALCL was confirmed by expert pathology review in each case. All testing was performed on fresh seroma fluid with adequate purity (>40%) of tumor cells confirmed by either cytological assessment or by multiparameter flow cytometry.

### Sequence variant, copy number change and structural variant detection by next generation sequencing (NGS)

Tumor samples were sequenced with the Peter MacCallum Cancer Centre (PMCC) PanHaem Panel as described previously [48]. Briefly, the PMCC PanHaem panel is a hybridisation based NGS panel targeting genes associated with haematological malignancy (Supplementary Table 1), the entire *IGH*/*TRA*/*TRB*/*TRG* and *TRD* loci and genome-wide copy number assessment. Two cases (BALCL7 and BALCL8) had undergone whole exome sequencing with the variant and copy number findings described previously [10].

### T-cell repertoire sequencing

T-cell receptor beta (*TRB*) loci deep amplicon sequencing was performed using LymphoTrack *TRB* (Invivoscribe, San Diego, CA, USA) as per manufacturer's instructions. Sequence assembly from FASTQs, annotation and error correction was performed by MiXCR [49] with secondary analysis (diversity assessment, VDJ family usage analysis) performed by VDJtools (ver 1.1.9) [50]. T-cell repertoire diversity was assessed by the normalized Shannon-Wiener entropy calculated as -Σ_i_(p_i_Lnp_i_)/LnN (where p_i_ is the frequency of each species and N is the total number of the species).

### Immunohistochemistry

For RANK immunohistochemical analysis, formalin-fixed paraffin embedded sections from BIA-ALCL patients were dewaxed in xylene prior to rehydration using graded ethanol concentrations. Antigen retrieval and immunostaining were performed as described [51] using monoclonal mouse anti-human RANK antibody (N-1H8; Amgen Inc.). For PDGFRA immunohistochemical analysis, monoclonal mouse anti-PDGFR-α (C-9) (Santa Cruz, sc-398206, dilution 1:100) was incubated overnight at 4°C following antigen retrieval with Dako Low pH 3 in 1 buffer at 97°C for 20 minutes in a Dako PT Link (Agilent Technologies, K8005). Visualization was performed on a Dako Autostainer48 using Envison Flex detection kit (Agilent Technologies, K5007).

## SUPPLEMENTARY MATERIALS FIGURES AND TABLES




