# Supplementary material for: Frequent activating STAT3 mutations and novel recurrent genomic abnormalities detected in breast implant-associated anaplastic large cell lymphoma
Source: Oncotarget. 2018 Nov 16;9(90):36126–36. doi: 10.18632/oncotarget.26308 (PMC6281423; doi:10.18632/oncotarget.26308)
Supplement: Supplementary file 1 [file oncotarget-09-36126-s001.pdf]

Frequent activating STAT3 mutations and novel recurrent genomic abnormalities detected in breast implant-associated anaplastic large cell lymphoma

SUPPLEMENTARY MATERIALS

Supplementary Table 1: Genes analysed by targeted sequencing. See Supplementary_Table_1
